# The Opposing Roles of Cellular Inhibitor of Apoptosis Proteins in Cancer

**DOI:** 10.5402/2012/928120

**Published:** 2012-08-09

**Authors:** R. Lau, M. A. C. Pratt

**Affiliations:** Breast Cancer Research Lab, Department of Cellular and Molecular Medicine, University of Ottawa, 451 Smyth Road, Ottawa, ON, Canada K1H 8M5

## Abstract

Cellular inhibitors of apoptosis proteins 1 and 2 (cIAP1/2) are members of the inhibitor of apoptosis protein (IAP) family that has been implicated in the pathology of human cancers due to their overexpression and function as blockers of cell death in various cancers. As a result, small molecule IAP antagonists have been developed and are currently under clinical evaluation for potential therapeutic use. In contrast, recent evidence has indicated a tumour-suppressing role for the cIAPs. Mutations in or loss of cIAPs have been identified as molecular lesions that contribute to constitutive activation of NF-**κ**B in hematopoietic malignancies. These studies reveal a context-dependent role for the cIAPs wherein both their overexpression and loss may contribute to tumourigenesis.

## 1. Cellular Inhibitor of Apoptosis Proteins ****(cIAPs)

The inhibitor of apoptosis proteins (IAPs) are potent suppressors of apoptosis and the human family is comprised of eight members: cellular IAP 1 (cIAP1), cellular IAP 2 (cIAP2), X-linked IAP (XIAP), neuronal apoptosis inhibitory protein (NAIP), melanoma IAP (ML-IAP), survivin, Apollon, and IAP like protein 2 (ILP2) [1]. All IAP proteins are characterized by the presence of one to three baculovirus IAP repeat (BIR) domains, which are zinc-binding regions of approximately 70 amino acids that mediate protein-protein interactions [2]. A number of IAPs also contain a RING (really interesting new gene) domain that confers ubiquitin protein ligase (E3) activity and are capable of auto-ubiquitination, as well as ubiquitination of proteins involved in apoptosis and signaling [3].

cIAP1 and cIAP2 can bind caspases, but do not directly inhibit them [4]. Instead, they exert their antiapoptotic effects through protein-protein interactions and by modulating the levels of other proteins through their function as ubiquitin ligases. Firstly, cIAP1/2 can bind to Smac and sequester it from XIAP, allowing XIAP to inhibit caspases and suppress apoptosis [5]. Furthermore, the cIAPs can target caspases and Smac for degradation by mediating their ubiquitination [5, 6]. The ubiquitin ligase activity of the cIAPs is conferred by the presence of the RING domain in their carboxy terminus and their substrates include themselves and proteins involved in signaling [7], including multiple substrates in the tumour necrosis factor receptor (TNFR) complex [8, 9]. This function imparts a role for cIAPs in the regulation of NF-*κ*B activation.

## 2. Regulation of NF-kB by cIAPs

 Activation of NF-*κ*B signaling regulates a large number of genes involved in a wide range of biological functions including cytokines, adhesion molecules, chemokines, and a number of genes that contribute to survival by promoting proliferation and inhibiting apoptosis [10].

**Figure 1:**
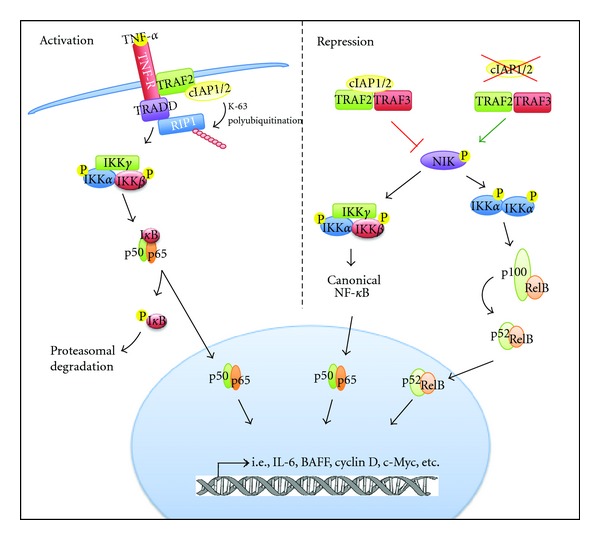
cIAP1/2 participates in positive and negative regulation of NF-*κ*B. cIAP1/2 are recruited to an activated TNF receptor where they mediate K-63 polyubiquitination of RIP1. RIP1 subsequently activates the IKK complex, resulting in activation of canonical NF-*κ*B complexes. In contrast, cIAP1/2 represses activation of canonical and noncanonical NF-*κ*B signaling by ubiquitinating NIK, leading to its degradation. Mutation or loss of cIAP1/2 results in accumulation of NIK, resulting in activation of both canonical and noncanonical NF-*κ*B. Activated NF-*κ*B complexes promote the transcription of various growth and survival factors, such as IL6 (Interleukin 6) and BAFF (B-cell activating factor). cIAP, cellular inhibitor of apoptosis; IKK, I*κ*B kinase; NIK, NF-*κ*B inducing kinase; RIP, receptor interacting protein; TNF, tumour necrosis factor; TNFR, TNF receptor; TRAF, TNFR-associated factor; TRADD, TNFR-associated death domain.

The cIAPs can regulate the canonical and noncanonical NF-*κ*B pathways in contrasting ways (Figure 1). cIAP1/2 plays a critical role in TNF receptor (TNFR) signaling to canonical NF-*κ*B [11]. Binding of TNF-*α* to TNFR induces the formation of complex I, consisting of TRADD (TNF receptor-associated death domain), TRAF2 (TNF receptor-associated factor 2), and RIP1 (receptor interacting protein 1) [12]. TRAF2 recruits cIAP1/2 to the complex, where they are required for TNFR-induced activation of NF-*κ*B signaling. The RING domains of cIAP1/2 catalyze the activating K63-linked polyubiquitination of RIP1, which activates the TAK1 (transforming growth factor-*β*-activated kinase 1) kinase complex [13]. This complex mediates the phosphorylation of IKK, which in turn phosphorylates I*κ*B to signal its degradation and activates canonical NF-*κ*B [14]. The K63-linked polyubiquitination of RIP1 also suppresses the activation of caspase-8 and formation of the proapoptotic complex II [15], thereby preventing apoptosis. The expression of prosurvival genes stimulated by TNF-*α* activation of NF-*κ*B signaling is believed to play a major role in the protection against TNF-*α*-induced cell death [16]. 

In contrast, cIAP1/2 can also repress NF-*κ*B activity. As previously mentioned, cIAP1/2 participate in a multi-subunit ubiquitin ligase complex that includes TRAF2 and TRAF3. This complex targets NIK and tonically represses it to limit the activation of both the canonical and noncanonical NF-*κ*B signaling [17–19]. Mutations in the constituents of this complex, including cIAP1/2, lead to constitutive activation of NF-*κ*B [20, 21]. 

Thus, the cIAPs can both positively and negative regulate the NF-*κ*B pathway. While cIAPs participate in the activating ubiquitination of RIP to result in activation of the canonical pathway in response to TNF/death ligands, they conversely ubiquitinate NIK in an inhibitory manner to suppress the both the canonical and noncanonical pathways.

## 3. Oncogenic Role of cIAPs

True to its name, the cIAPs play an important role in the inhibition of apoptosis. They are induced to promote survival during cellular stresses such as detachment from extracellular matrix [22] and ER stress [23]. They are also induced by pro-survival signaling such as nuclear factor (NF)-*κ*B [24, 25]. Not surprisingly, their antiapoptotic activity is exploited for tumour cell survival, and their expression is induced by potent oncogenes such as Ras [26] and E6 [27]. Many members of the IAP family, including the cIAPs, are overexpressed in a number of human cancers and are associated with poor prognosis [28]. Direct genetic evidence has demonstrated the cIAPs as protooncogenes. Chromosomal amplification of the 11q21–23 region, which encompasses both cIAP1 and cIAP2, is observed in a variety of cancers, including renal cell carcinomas, glioblastomas, gastric carcinomas, and nonsmall cell lung carcinomas [29–31]. Additional genetic evidence comes from MALT (mucosa-associated lymphoid tissue) lymphoma. Approximately 50% of surveyed cases displayed a *t*(11,18) (q21; q21) translocation, which results in a fusion of the BIR domains of cIAP2 with the carboxy terminus of the paracaspase domain of MALT1 [32, 33]. The resulting fusion protein promotes constitutive activation of NF-*κ*B, leading to increased prosurvival signaling. 

### 3.1. IAP Antagonists

The anti-apoptotic function of IAPs and their overexpression in a wide variety of cancers make them attractive therapeutic targets. As such, a number of strategies to target IAP proteins in cancer are currently under investigation. One focus has been on the generation of molecules that mimic the aminoterminus of mature Smac. These Smac mimetics disrupt IAP : caspase and IAP : SMAC interactions and can stimulate cell death [34]. Originally designed to target XIAP, these antagonists exhibit higher affinities for the cIAPs, triggering autoubiquitination and proteasomal degradation. Following treatment with an IAP antagonist, it is speculated that the downregulation of cIAPs result in the accumulation of NIK, which activates noncanonical NF-*κ*B signaling and leads to autocrine TNF-*α* production [35–37]. In the absence of cIAPs, activation of survival genes by p65/RelA is greatly reduced [14, 38]. TNF-*α* instead triggers the formation of the pro-apoptotic complex II, consisting of FADD, caspase-8, and deubiquitinated RIP1, resulting in apoptosis [39]. While treatment with IAP antagonists as a single agent has shown some success in a limited number of human cancer cell lines, their cytotoxicity is often augmented when used in combination with other agents such as TNF-*α* and tumour necrosis factor-related apoptosis-inducing ligand (TRAIL) [30, 40].

## 4. Tumour Suppressing Role of cIAPs

While overexpression of cIAPs likely promote tumourigenesis by inhibiting apoptosis through their interactions with components of the apoptotic machinery (i.e., SMAC/DIABLO), the loss of cIAPs in a number of blood malignancies is associated with pro-survival activation of NF-*κ*B signaling. Recent studies have shown that mutations or translocations resulting in the loss of cIAPs are, in part, responsible for constitutive activation of NF-*κ*B signaling in several cancers. Two independent groups reported that constitutive activation of NF-*κ*B signaling in multiple myeloma may be attributable to alterations in cIAP2 or components of the NIK-regulating complex including cIAP1, TRAF2, and/or TRAF3 [20, 21]. The resulting NIK-mediated activation of both canonical and noncanonical NF-*κ*B signaling is essential for promoting tumour cell survival in multiple myeloma [20]. Aberrant activation of NF-*κ*B is also detected in a majority (~60%) of splenic marginal zone lymphomas (SMZL) [41]. Investigation into possible molecular lesions in the NF-*κ*B pathway revealed disruption of cIAP2 as a contributor to constitutive NF-*κ*B activation. Out of 101 SMZL cases analyzed, 11% of cases harboured abnormalities in cIAP2 by inactivating mutations, missense mutations and gene deletions. These mutations were all monoallelic, suggesting that genetic lesions in cIAP2 may have a dominant negative effect. All of the SMZL primary cases displaying cIAP2 mutations showed constitutive activation of NF-*κ*B signaling, including accumulation of NIK. 

Rossi et al. recently showed that genetic disruption of cIAP2 in chronic lymphocytic leukemia is associated with fludarabine resistance and a poor outcome similar to that attributed to *TP53* abnormalities [42]. Additionally, progressive but fludarabine-sensitive disease was devoid of cIAP2 mutations/loss, indicating that the genetic lesions are specifically associated with a chemoresistant phenotype. The loss of cIAP2 in these lesions was found to be associated with constitutive activation of NF-*κ*B, consistent with its role as a negative regulator of NIK. Interestingly, disruption of cIAP2 through inactivating mutations and/or gene deletions was found to be mutually exclusive with *TP53* abnormalities. 

Since NF-*κ*B is a known negative regulator of p53 [43], it is possible that the loss of cIAP2 in these cells results in reduced p53 function, thus obviating the requirement for inactivating mutations in *TP53*. In line with this, our lab has shown that in breast mammary epithelial cells, the downregulation of cIAP2 results in reduction of wild-type p53 protein (unpublished data). This may also explain why CLL patients lacking functional cIAP2 display a similar outcome as patients with *TP53* abnormalities.

## 5. Conclusion

Transient activation of NF-*κ*B signaling is utilized by normal B-lymphocytes to promote cell survival and differentiation as a response to antigens [44]. However, aberrant activation of NF-*κ*B is a major contributor to the oncogenesis [45]. The loss of cIAPs in a number of lymphoid malignancies results in constitutive activation of both canonical and non-canonical NF-*κ*B signaling, leading to increased survival and proliferative signals. In contrast to the oncogenic role typically attributed to IAPs, these recent studies have shown a tumour-suppressing role for the cIAPs in limiting NF-*κ*B activity. These studies underscore the importance of the cellular context under which cIAPs are therapeutically targeted since overexpression and loss can both contribute to cancer cell progression.
